# A systematic review of features and content quality of Arabic mental mHealth apps

**DOI:** 10.3389/fdgth.2024.1472251

**Published:** 2024-12-11

**Authors:** Noorah Ibrahim S. Alnaghaimshi, Mona S. Awadalla, Scott R. Clark, Mathias Baumert

**Affiliations:** ^1^ Discipline of Biomedical Engineering, School of Electrical and Mechanical Engineering, The University of Adelaide, Adelaide, SA, Australia; ^2^ Department of Computer and Information Science, Al-Majmaah University, Al-Majmaah, Riyadh Region, Saudi Arabia; ^3^ Specialist Community Mental Health Services, Southern Adelaide Local Health Network, Adelaide, SA, Australia; ^4^ Discipline of Psychiatry, School of Medicine, The University of Adelaide, Adelaide, SA, Australia

**Keywords:** m-Health, mental health, Arabs, Arabic apps, depression, anxiety

## Abstract

**Introduction:**

Anxiety and depression are major causes of disability in Arab countries, yet resources for mental health services are insufficient. Mobile devices may improve mental health care delivery (mental m-Health), but the Arab region's mental m-Health app landscape remains under-documented. This study aims to systematically assess the features, quality, and digital safety of mental m-Health apps available in the Arab marketplace. We also contrast a set of recommended Australian apps to benchmark current strategies and evidence-based practices and suggest areas for improvement in Arabic apps.

**Methods:**

Fifteen Arab country-specific iOS Apple Stores and an Android Google Play Store were searched. Apps that met the inclusion criteria were downloaded and evaluated using the Mobile App Rating Scale (MARS) and the Mobile App Development and Assessment Guide (MAG).

**Results:**

Twenty-two apps met the inclusion criteria. The majority of apps showed no evidence of mental health experts being involved in the app design processes. Most apps offered real-time communication with specialists through video, text, or audio calls rather than evidence-based self-help techniques. Standardized quality assessment showed low scores for design features related to engagement, information, safety, security, privacy, usability, transparency, and technical support. In comparison to apps available in Australia, Arabic apps did not include evidence-based interventions like CBT, self-help tools and crisis-specific resources, including a suicide support hotline and emergency numbers.

**Discussion:**

In conclusion, dedicated frameworks and strategies are required to facilitate the effective development, validation, and uptake of Arabic mental mHealth apps. Involving end users and healthcare professionals in the design process may help improve app quality, dependability, and efficacy.

## 1 Introduction

Mental health problems significantly impact people's lives globally, with the total disability-adjusted life years (DALYs) from mental disorders increasing from 80.8 million in 1990 to 125.3 million in 2019 (1). Arab nations are not an exception, and depression and anxiety are among the top causes of disability (2). Public expenditure per capita in the Eastern Mediterranean Region (US$2) is well below spending in Europe (US$23) and the Americas (US$12) (3), and access to professional care is poor (4).

Three major barriers to Arab migrants and refugees seeking mental health treatment have been identified: a low mental health literacy level, a lack of awareness of available resources, and stigma and social discrimination (5). Data on the engagement of Arab people with mental health services is limited (6), with most Arabs, in both Arabic and Western settings, holding a negative attitude towards formal mental health services and help-seeking (7–9). Arab traditions may pose significant barriers to seeking formal diagnosis and treatment (10). Cultural beliefs, social taboos, stigma, and suspicions around mental health represent dominating influences on public perception of mental disorders (11–13). Indeed, mental health and psychological service delivery models can be critical to Arabs' perception of mental disorders and willingness to seek care. Internet-delivered interventions could provide anonymity to deal with the stigma and burden of mental illness (14). Studies show the majority of Arab people would prefer digital mental health intervention over face-to-face consultation (15, 16).

Mobile health (mHealth), defined as medical and public health care utilizing mobile devices like mobile phones, patient-monitoring devices, digital assistants, and other wireless devices, can address the need for accessible, affordable, scalable, culturally acceptable mental health care (17, 18). Globally, smartphone ownership and Internet use are increasing, and 70% of Arabs use the Internet (19), creating an opportunity for the uptake of mHealth apps. mHealth apps are becoming a standard part of care in Western countries, acting as “a digital lifeline” (20).

Recent reviews suggest mHealth apps can effectively reduce symptoms of mental health disorders, including depression and anxiety (21, 22). They can be targeted to different stages of clinical care delivery, from enhancing access to improving evidence-based treatment outcomes (23). mHealth apps can assist in diagnosing and managing mental health disorders in several ways. The ability to integrate digital data from activities, cognitions, and behaviours using survey scores (ecological momentary assessments), cognitive test scores, and environmental and social context tagging can provide relevant clinical insights used to develop personalized treatment strategies (24). mHealth apps may help users identify and differentiate emotions (25), and assist in restoring their emotional equilibrium during distress (26). Additionally, mHealth apps may enhance patient awareness of their mental health issues, educate them about treatment processes before seeking mental health care and provide advice on the best available practice (23).

While mHealth apps have clear benefits, they should be evaluated to verify their efficacy and safety given the risks associated with severe mental illness and the potential for interventions to be ineffective or even to do harm (27, 28). App evaluation can occur at several levels, including user-based app marketplace evaluation, e.g., star ratings and user reviews, and expert review platforms such as PsyberGuide or RankedHealth (29). Evaluation tools using clinical standards and guidelines include the Mobile App Rating Scale. However, these assessments can be challenging for non-experts and less practical for clinicians and patients to make immediate decisions (29). The American Psychiatric Association have developed a patient-centric app evaluation framework, offering practical guidance for clinicians to ensure app safety, effectiveness, and usefulness in clinical settings (29). While such app evaluation frameworks may drive design and improve usability and validity over time, no specific regulatory processes exist internationally unless the app is linked to or takes the place of recognized medical devices (akin to software as a medical device within the FDA regulatory framework) (28).

To obtain a detailed understanding of the landscape of the Arab region's mental mHealth apps, we systematically reviewed and analyzed their quality and safety using validated quantitative and qualitative tools and compared them with popular Australian apps, recommended by the government and prominent non-government bodies.

## 2 Method

### 2.1 Systematic search of the Arabic marketplace

**Figure 1:**
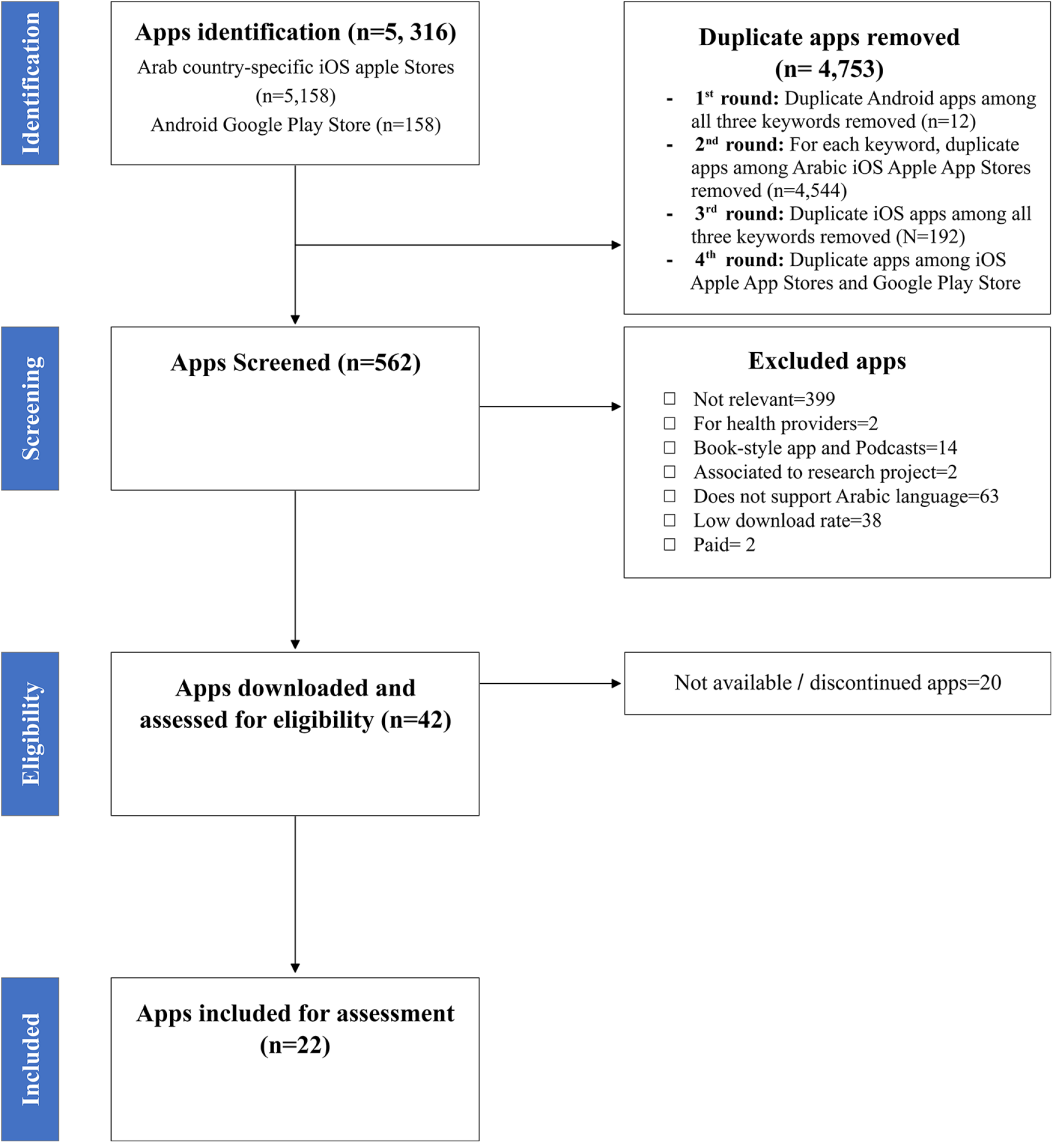
Flowchart for searching and selecting Arabic mental mHealth apps.

Between April and May 2023, the author NA systematically searched relevant mental mHealth apps in the Arab marketplace following established criteria (30, 31) (see Figure 1). We considered apps publicly available for download on the two most popular mobile phone platforms, the Arabic Apple App Stores and Android Market (Google Play), targeted at the individual adult mental health consumer that were free to download and freemium apps.

At the time of our wide-ranging search, we identified only 15 Arab country-specific iOS Apple Stores available across recognized Arabic countries (Jordan, Egypt, Tunisia, Algeria, Lebanon, Yemen, Mauritania, KSA, Qatar, Bahrain, UAE, Kuwait, Oman, Iraq, and Morocco). We then searched country-specific Apple stores and the Android Google Play Store via AppBrain, by Arabic using three general Arabic search terms related to mental health: “Mental health” (الصحة العقلية), “psychological health” (الصحة النفسية), and “psychotherapy” (العلاج النفسي).

Duplicate apps across search terms, the 15 country-specific stores, Android Google Play Store and iOS Apple stores were removed. Author NA reviewed the remaining apps using predefined inclusion/exclusion criteria. In uncertain cases, app inclusion or exclusion was verified with SRC and MB. We excluded apps that were not relevant to mental health care and service, did not support the Arabic language, were not designed to treat or assess a mental health condition, book library apps, book-style apps, podcast apps, those targeted at the health providers and professionals, those designed for children, paid apps, and apps that were associated with a research project and apps with low download rate (<100). AppBrain download rates were used to determine Android app dissemination. A third-party tool (ASOTools^1^) was used to track download rates for iOS apps in August 2023 since download rates for the Apple App Store are not publicly available. The resulting apps were divided into broad categories based on their descriptions in the storefront criteria (Table 1). Following this initial screening, all remaining apps were downloaded for assessment. During this step, 20 apps were excluded due to discontinuation or unavailability. Figure 1 shows the PRISMA diagram documenting these processes.

**Table 1:** Summary of the Arabic mental mHealth apps included in this review.

No.	App name in Arabic	App name in English	Key word/s	Platform	App category	Country-specific stores (i.e., for iOS apps)	Target mental health concern(s)	Affiliations
1	استنارة	Estenarh	العلاج النفسي	iOS	Medical	Jordan, Egypt, Tunisia, Algeria, Lebanon, Yemen, KSA, Qatar, Bahrain, UAE, Kuwait, Oman, Iraq, Morocco.	Targeted at a variety of disorders, including anxiety, depression, obsessive-compulsive disorder, gender dysphoria, mood disorder, and others.	Commercial
2	شيزلونج	Shezlong	الصحة النفسية	iOS	Medical	Jordan, KSA, Qatar, Kuwait, Oman, UAE, Bahrain, Egypt, Lebanon, Tunisia, Algeria, Mauritania, Iraq, Yemen, Morocco.	Targeted at a variety of disorders, including anxiety, depression, personality disorders, eating disorders, and others.	Commercial
3	O7 ثيرابي	O7 Therapy	الصحة العقلية، الصحة النفسية	iOS	Health & Fitness	Jordan, Qatar, Kuwait, Oman, UAE, Bahrain, Egypt, Morocco.	Targeted at a variety of disorders, including anxiety, depression, couples’ therapy, and others.	Commercial
4	لبيه	Labayh	الصحة النفسية	iOS	Medical	Jordan, KSA, Qatar, Kuwait, Oman, UAE, Bahrain, Egypt, Lebanon, Tunisia, Algeria, Mauritania, Iraq, Yemen, Morocco.	Targeted at a variety of disorders, including anxiety, depression, schizophrenia, social phobia, gender identity disorder, and others.	Commercial
5	أيادي	Ayadi | therapy & counselling	الصحة العقلية، الصحة النفسية، العلاج النفسي	iOS	Health & Fitness	Jordan, Egypt, Tunisia, Algeria, Lebanon, Yemen, Mauritania, KSA, Qatar, Bahrain, UAE, Kuwait, Oman, Iraq, Morocco.	Targeted at a variety of disorders, including anxiety, depression, social phobia, and others.	Commercial
6	تطمن - استشارات نفسية	Tetaman	العلاج النفسي، الصحة النفسية	iOS	Medical	KSA, Iraq, Morocco.	Targeted at a variety of disorders, including anxiety, depression, social phobia, obsessive-compulsive disorder, and others.	Commercial
7	المرشد	AlMorshed	العلاج النفسي	iOS & Android	Medical	Jordan, Egypt, Tunisia, Algeria, Lebanon, Yemen, Mauritania, KSA, Qatar, Bahrain, UAE, Kuwait, Oman, Iraq, Morocco.	Targeted at a variety of disorders, including anxiety, depression, personality disorders, couples’ therapy, and others.	Commercial
8	مايند	Mind	الصحة النفسية	iOS & Android	Business	Jordan, KSA, Qatar, Kuwait, Oman, UAE, Bahrain, Egypt, Lebanon, Tunisia, Algeria, Mauritania, Iraq, Yemen, Morocco.	Targeted at a variety of disorders, including anxiety, depression, gender dysphoria, mood disorder, and others	Commercial
9	فسرلي	Faserly	الصحة النفسية	Android	Lifestyle	NA	Targeted at a variety of disorders, including anxiety, depression, couples’ therapy, and others.	Commercial
10	داعم	Daeim	الصحة النفسية	Android	Medical	NA	Targeted at a variety of disorders, including anxiety, depression, couples’ therapy, and others.	Commercial
11	كيورا	Cura	الصحة النفسية	iOS	Medical	KSA	Targeted at a variety of disorders, including anxiety, depression, and others.	Commercial
12	سنار	Sanar	الصحة العقلية، الصحة النفسية	iOS	Medical	Jordan, KSA, Qatar, Kuwait, Oman, UAE, Bahrain, Egypt, Lebanon, Tunisia, Algeria, Mauritania, Iraq, Yemen, Morocco.	Targeted at a variety of disorders, including anxiety, depression, obsessive-compulsive disorder, mood disorder, and others.	Commercial
13	الشخصية اختبار النرجسية	Akhtibar Alshakhsia Alnarjisia	الصحة العقلية	Android	Entertainment	NA	Narcissistic personality disorder	Unknown
14	- شفاء المؤمن الرقية الشرعية	Alruqayat Alshareia	العلاج النفسي	iOS	Books	Jordan, Egypt, Tunisia, Algeria, Lebanon, Yemen, Mauritania, KSA, Qatar, Bahrain, UAE, Kuwait, Oman, Iraq, Morocco.	Not specified	Unknown
15	الصحة النفسية	Alsiha Alnafsia	الصحة النفسية	Android	Education	NA	Not specified	Unknown
16	العقلية الصحة النفسية و	Alsiha Alnafsia w aleaqlia	الصحة النفسية، الصحة العقلية	Android	Health & Fitness	NA	Stress, anxiety, depression	Unknown
17	دليلك النفسي	Daliluk Alnafsi	العلاج النفسي، الصحة النفسية	iOS & Android	Health & Fitness	KSA	Not specified	NCMH
18	توكيدات : ايجابية امتنان راحة	Tawkidat	الصحة النفسية	iOS & Android	Health & Fitness	Jordan, KSA, Qatar, Oman, UAE, Bahrain, Egypt, Lebanon, Tunisia, Algeria, Mauritania, Iraq, Yemen, Morocco.	Not specified	Commercial
19	نفس	Nafas	الصحة النفسية	iOS	Health & Fitness	Jordan, KSA, Qatar, Kuwait, Oman, UAE, Bahrain, Egypt, Lebanon, Tunisia, Algeria, Mauritania, Iraq, Yemen, Morocco.	Anxiety and stress	Commercial
20	تهون	Tuhoon	الصحة النفسية	iOS	Health & Fitness	Jordan, KSA, Qatar, Kuwait, Oman, UAE, Bahrain, Egypt, Lebanon, Tunisia, Algeria, Mauritania, Iraq, Yemen, Morocco.	Anxiety, stress, well-being	Commercial
21	كن	Kun Being: Workforce Wellness	الصحة العقلية، الصحة النفسية	iOS	Health & Fitness	Jordan, Mauritania, Iraq, Morocco	Depression, stress, and anxiety	Commercial
22	توازن	Tawazon	الصحة النفسية	iOS	Health & Fitness	Jordan, KSA, Qatar, Kuwait, Oman, UAE, Bahrain, Egypt, Lebanon, Tunisia, Algeria, Mauritania, Iraq, Yemen, Morocco.	Anxiety and stress	Commercial

### 2.2 Selection of Australian mental health apps

We compared the quality of mental health apps from the Arabic region with four mental mHealth apps currently accessible in the Australian market. We chose Smiling Mind, ReachOut WorryTime, HeadGear, and MoodMission based on government-funded agency recommendations (i.e., Head to Health, Health Direct, and ReachOut), user ratings, target group, cost, and effectiveness. ReachOut WorryTime, HeadGear, and MoodMission use CBT strategies, while Smiling Mind is a mindfulness meditation app. All four apps are free to download and targeted towards the individual mental health consumer. HeadGear and MoodMission have undergone successful clinical trials (32–35). A randomized controlled trial found that HeadGear app significantly reduced depressive symptoms in the intervention group, with a lower prevalence of 3.5% over a 12-month period (34). Likewise, in a 30-day randomized controlled trial, the MoodMission app significantly improved mental well-being, reduced depression and increased coping self-efficacy. However, it did not significantly reduce anxiety levels and showed no significant changes in emotional self-awareness or mental health literacy (32).

### 2.3 Evaluation criteria and quality assessment procedure

During September and October 2023, the apps were evaluated by two independent Arabic-speaking reviewers (NA and MA). Apps were downloaded and installed on an Apple iPhone X and a Samsung Galaxy S21 to evaluate their content, and information on Android, Google Play, and iOS Apple App Stores was accessed. For apps with freemium versions, the freely available content was assessed. We used the Mobile App Rating Scale (MARS) (36), as well as the Mobile App Development and Assessment Guide (MAG) (37) to assess app quality. Disagreements in MARS and MAG scores between the reviewers were discussed with the other authors to establish a consensus.

MARS is a popular tool for assessing the quality of mHealth apps (38) and contains 23 items divided into five categories: engagement, functionality, aesthetics, information quality, and subjective quality. Reviewers rate the quality of the apps on a 5-point scale (1 = inadequate, 2 = poor, 3 = acceptable, 4 = good, 5 = excellent). Six app-specific items (section F), which can be customized to include or exclude relevant information on the subject of interest, were excluded to standardize the review. The MARS lacks items that consider app privacy and security, and it does not consider existing guidelines for health and medical equipment software development (37, 39). We also used the Mobile App Development and Assessment Guide (MAG) (37) to address these limitations. The MAG incorporates 48 binary items divided into eight categories: usability, privacy, security, appropriateness and suitability, transparency and content, safety, technical support and updates, and technology.

## 3 Results

### 3.1 Overview of included Arabic mental mHealth apps

We identified 5,316 apps from Arab country-specific iOS Apple Stores (*n* = 5,158) and Android Google Play Store (*n* = 158). After screening app titles and descriptions, 42 apps were downloaded for further assessment, of which 22 apps matched the inclusion criteria. We measured inter-rater agreement between the two independent reviewers who assessed all apps in the study, both Arabic and Australian apps. Each reviewer evaluated every app using the MARS and MAG subscales to provide a structured assessment across multiple criteria. Cohen's kappa was calculated using IBM SPSS Statistics V. 29, to account for the possibility of agreement occurring by chance, providing an objective measure of consistency between reviewers. An interrater agreement of kappa = .49 for the MARS indicates a moderate level of inter-rater reliability between the two reviewers, whereas kappa = .96 for the MAG, indicates a near-perfect level of inter-rater reliability, based on Landis and Koch's framework (40). The higher level of agreement for MAG can be explained by the fact that it has less subjective content than MARS, which could account for the difference in agreement levels between the two.

Table 1 shows the characteristics of the 22 Arabic apps in our analysis. Seventeen were developed by commercial entities and 12 of these apps (app 1–12) provided mental health counselling services delivered by health professionals (e.g., psychologists, psychotherapists, or psychiatrists). All apps offering counselling services could be downloaded for free, but with charges for counselling and could be further categorized as follows: Multipurpose health counselling, including mental health (*n* = 2); Mental health-only consultations (*n* = 9); and consultations in mental health and personal and professional development (*n* = 1). Four of the 17 commercial apps (app 19–22) were designed to facilitate guided intervention to relieve stress and anxiety and support well-being through breathing and meditation exercises. All mindfulness/meditation-based apps were developed adopting a “freemium” model, which allows users to access basic features for free and pay for premium features. One commercial app (app 18) was free and designed to employ positive affirmations to assist users in improving their thinking and emotions.

Four apps (app 13–16) were from unknown sources in terms of developer's identification, qualification, and whether these apps come from an accredited health organization. Two apps (apps 15 and 16) were designed solely to deliver information/education with minimum user interaction. However, the information provided in these apps did not indicate whether it came from a reliable source/s. One app (app 14) was of a religious/spiritual faith-based type and was designed to solely deliver intervention based on spiritual faith or religious practices, as a resource for coping with mental disorders and as a source of comfort and hope. Finally, one app (app 13) was designed solely as a self-assessment tool for identifying narcissistic personality traits. The app did not reference supporting literature or recommend specialist/doctor contact for expert assessment, guidance, and treatment if necessary.

Apps from reputable sources such as research centers, health institutions, and universities were lacking. Only one app (app 17) was available on the Saudi iOS Apple Store and developed in affiliation with The National Center for Mental Health Promotion (NCMH), Saudi Arabia. The app is intended to serve as a directory tool for all mental health service providers in the Kingdom. The app lists the psychological and support services provided to patients by each party.

### 3.2 Mental health App features

Supplementary Tables S1–S10 summarise the features of the Arabic mental mHealth apps reviewed. The most common feature was access to health professional support, followed by education and awareness components. Video-based counselling sessions were the most common method to deliver health professional support, followed by text and phone calls (Supplementary Tables S1). These apps enabled users to search and pay for one-on-one counselling sessions. Common features were tailored search filters for finding suitable mental health professionals according to needs (Supplementary Tables S2), alerting/reminder functions, and review and rating options.

The most common form of information/education in Arabic apps was plain text, with minimal visualization with charts or graphs (Supplementary Tables S3). Some also offered access to blogs with informative articles (in 5 apps; 2,4,7,8,9), podcasts (in 3 apps; 19,20,22) and online webinars/workshops/courses (in 3 apps; 3,4,8). Only one app offered social networking for support via the option to build a virtual identity for an app community (Supplementary Tables S4).

Only 4 of 22 apps (app 19–22) provided self-help strategies and techniques, all focussed on mindfulness/meditation/relaxation techniques to improve emotion regulation, stress coping, and relaxation (Supplementary Tables S5). In terms of culturally relevant content, notably, one app (app 20) offered a collection of guided meditations influenced by Islamic teachings and one app (app 14) was designed specifically to deliver audio-based recitation (Ruqyah), supplication (Duaa), and remembrance (Thiker).

Arabic apps lacked behavioural targeting, tracking mechanisms, and functionality for CBT-based processes such as behavioural activation or management of negative thoughts (Supplementary Tables S6 and S7). Only one app had a journaling feature (app 20), which required a paid subscription. Only one app (app 18) included interactive settings for positive affirmation selection, frequency, and list creation. Regarding mood/emotion tracking (Supplementary Tables S8), only three apps (app 4,20,22) collected information about users' current moods and provided a visual representation of mood state. Two of the three apps enquired about the reason for the current mood. These momentary assessments were simplistic, using simple questions with emojis that described mood or feeling.

By comparison, self-help tools and features that promote evidence-based strategies and techniques to manage anxiety and depression are prioritized in Australian apps. HeadGear and MoodMission were designed to assist users in developing skills to improve mental health and offer features that motivate users to engage in specific challenges or missions. MoodMission invites users to choose alternatives for activities/exercises for more engagement and a sense of control. Both apps employ gamification techniques and rewards to increase therapeutic commitment among users, a technique used by only a single Arabic app (app 20) to encourage users to complete guided meditation modules (Supplementary Tables S9). One of the apps (ReachOut WorryTime) was designed to prompt user to identify and respond to their worry thoughts by offering a worry thought record and features to set a time for reflections and duration of reflections, a worry thought count, and the option to erase recorded worry thoughts that no longer matter. The app is built in a way that is entirely based on user input and has no mechanism for providing feedback; further, it does not use questioning to help users evaluate their thoughts or worries. One Australian app (Smiling Mind) was designed to prompt meditation and mindfulness and employs similar features (such as guided audio modules, offline downloads, and meditation stats) found in Arabic apps designed for the same purpose.

### 3.3 MARS quality assessment scores

Table 2 details the quality assessment of Arabic apps using the MARS instrument. Based on total score, out of 22, five apps (23%) were classified as good (ranging from 4.12 to 3.88), ten (45%) as acceptable (ranging from 3.50 to 3.05), four (18%) as poor (ranging from 2.88 to 2.55), and three (14%) as inadequate (ranging from 1.65 to 1.48).

**Table 2:** Mobile application rating scale (MARS) ratings of the Arabic and Australian mental health apps.

No.	Application	Engagement	Functionality	Aesthetics	Information	Subjective quality	MARS (mean)
Arabic apps							
1	Estenarh	2.90	4.50	3.50	3.30	1.63	3.14
2	Shezlong	2.90	4.13	3.33	3.20	2.13	3.12
3	O7 Therapy	3.60	4.75	4.17	3.60	3.50	3.88
4	Labayh	4.10	4.50	4.17	3.60	3.25	3.90
5	Ayadi	2.90	4.25	3.33	3.10	2.00	3.10
6	Tetaman	3.10	4.25	3.67	3.10	2.50	3.29
7	AlMorshed	2.80	4.13	3.33	3.38	2.63	3.23
8	Mind	3.30	4.50	4.00	3.30	2.63	3.50
9	Faserly	2.70	3.75	3.17	2.75	2.13	2.88
10	Daeim	2.70	3.63	3.17	2.75	1.63	2.75
11	Cura	2.80	4.25	3.33	3.20	2.38	3.17
12	Sanar	2.80	4.38	3.33	3.40	2.38	3.24
13	Akhtibar Alshakhsia Alnarjisia	1.40	3.00	1.17	1.07	1.00	1.52
14	Alruqayat Alshareia	2.40	3.88	2.83	3.17	3.00	3.05
15	Alsiha Alnafsia	1.30	2.75	1.50	1.50	1.00	1.65
16	Alsiha Alnafsia w aleaqlia	1.20	3.13	1.00	1.07	1.00	1.48
17	Daliluk Alnafsi	2.40	3.25	2.50	3.21	1.75	2.79
18	Tawkidat	2.10	3.50	3.33	2.29	1.50	2.55
19	Nafas	3.60	4.88	4.67	3.79	4.25	4.12
20	Tuhoon	4.00	4.13	4.83	3.71	4.25	4.07
21	Kun Being	3.20	4.00	3.67	3.21	3.00	3.40
22	Tawazon	3.60	4.38	4.67	3.50	4.25	3.93
Australian apps							
	HeadGear	4.00	4.63	4.33	4.43	4.88	4.43
	ReachOutWorryTime	2.40	4.00	4.17	3.50	1.88	3.08
	Smiling Mood	4.00	4.75	4.17	4.00	3.63	4.09
	MoodMission	3.80	4.25	3.00	4.07	4.25	3.93

Of the Arabic mindfulness/meditation apps (app 19,20,22) achieved the highest overall score (4.12, 4.07, and 3.93, respectively), whereas (app 21) received an acceptable 3.40. Out of 12 Arabic apps providing online counselling, 2 (app 3 and 4) were classified as good, eight as acceptable, and two as poor in terms of overall mean quality, whereas apps that were solely meant to deliver information/education (app 15 and 16) were all rated as inadequate.

Overall, Arabic apps scored highest in functionality and aesthetics subscales. Fourteen of 22 apps (app 1–8,11,12, and 19–22) were rated as good on the functionality subscale, including learning, navigation, flow logic, and gestural design, seven were acceptable, while one was poor (app 15). Six apps (app 3,4,8,19,20,22) reached good scores on the aesthetics subscale, including graphic design, overall visual appeal, colour scheme, and stylistic consistency, 11 (app 1,2,5–7,9–12,18,21) obtained acceptable, two poor (app 14 and 17), and three (app 13,15,16) inadequate scores. However, for most of these apps' subjective quality, engagement, and information subscales were low. Only two apps (app 4,20) were classified as good for engagement, which included being fun, engaging, customizable, interactive, and well-targeted to the audience. Six (app 3,6,8,19,21,22) were acceptable, 11 were poor (app 1,2,5,7,9–12,14,17,18), and three (app 13,15,16) were inadequate. Regarding information quality, 16 apps (app 1–8, 11, 12, 14, 17, 19–22) were assessed as acceptable, three as poor (app 9,10,18), and three as insufficient (app 13,15,16). Of the 22 Arabic apps, three (app 19,20,22) were classified as good, four (app 3,4,14,21) as acceptable, eight (app 2,5–9,11,12) as poor, and seven (app 1,10,13,15–18) achieved inadequate subjective quality scores, which included not being worth recommending, not stimulating repeat use and poor overall satisfaction rating.

Among the Australian apps, HeadGear achieved the highest overall score on MARS (4.43), followed by Smiling Mood (4.09), MoodMission (3.93), and ReachOutWorryTime (3.08) (Table 2). Australian apps outperformed Arabic apps on MARS subscales in functionality and information quality, with all apps classified as good in functionality. Australian apps are highly responsive, and user-friendly, and users can seamlessly navigate the app, making interactions feel natural and constant throughout. Three apps were classified as good, and one (ReachOutWorryTime) was considered acceptable for information quality. In general, Australian apps have clear goals, high-quality, well-balanced content, and are from credible sources with a strong evidence base. Australian apps also outperformed MARS subscales in aesthetics, with 3 out of 4 apps achieving good scores, while one (MoodMission) was acceptable. Similarly, the engagement scores of three apps are classified as good, while one was poor (ReachOutWorryTime). Nonetheless, two apps are classified as good (HeadGear, MoodMission), one as acceptable (Smiling Mood), and one (ReachOutWorryTime) as having inadequate subjective quality scores.

### 3.4 MAG quality assessment scores

Table 3 shows that 86% of Arabic apps (*n* = 22) scored below 0.5, with overall MAG scores ranging from 0.13 to 0.6. Only 3 out of 12 Arabic apps (app1–3) offering online counselling received a moderate score of overall mean quality of 0.5 or higher. In contrast, the remaining (9/12) received a below-moderate score (ranging from 0.25 to 0.47), indicating a range of potential limitations and challenges in those apps' design that may pose risks to users, primarily related to safety and security. Despite receiving the highest overall score on MARS, Arabic mindfulness/meditation apps (app 19,20,21,22) receive a moderate overall score on MAG (ranging from 0.44 to 0.52), owing notably to low scores in the safety, security, usability, and technical support subscales. Apps focused on mental health education (app 15 and 16) had low MAG scores of 0.28 and 0.13.

**Table 3:** Mobile App development and assessment guide (MAG) ratings of the Arabic and Australian mental mHealth apps.

No	Application	Usability	Privacy	Security	Appropriateness	Transparency and content	Safety	Technical support	Technology	MAG (mean)
Arabic apps										
1	Estenarh	0.5	0.68	0.61	0.5	1	0.29	0.5	0.75	0.58
2	Shezlong	0.5	0.61	0.56	0.5	1	0.29	0	0.75	0.53
3	O7 Therapy	0.5	0.82	0.67	0.5	1	0.07	0.5	0.75	0.6
4	Labayh	0.5	0.64	0.33	0.5	0.5	0	0.5	0.75	0.46
5	Ayadi	0.5	0.54	0.56	0.5	0.5	0	0.5	0.75	0.47
6	Tetaman	0.5	0.39	0.33	0.5	0.5	0	0.5	0.75	0.39
7	AlMorshed	0.5	0.61	0.33	0	0.5	0	0	0.75	0.41
8	Mind	0.5	0.32	0.33	0.5	0.5	0	0	0.75	0.34
9	Faserly	0.5	0.61	0.33	0	0.5	0	0.5	0.75	0.43
10	Daeim	0.5	0.07	0.33	0	0.5	0	0	0.75	0.25
11	Cura	0.5	0.32	0.33	0.5	0.5	0	0.5	0.75	0.36
12	Sanar	0.5	0.54	0.44	0.5	0.5	0.14	0.5	0.75	0.47
13	Akhtibar Alshakhsia Alnarjisia	0.38	0	0	0.5	0	0	0	0.75	0.15
14	Alruqayat Alshareia	0.56	0	0	0	0.5	0	0	0.75	0.18
15	Alsiha Alnafsia	0.5	0.32	0.22	0	0	0	0	0.75	0.28
16	Alsiha Alnafsia w aleaqlia	0.38	0	0	0	0	0	0	0.75	0.13
17	Daliluk Alnafsi	0.56	0.32	0.17	1	0.25	0.29	0	0.75	0.38
18	Tawkidat	0.63	0.5	0.33	0.5	0	0	0.5	0.75	0.42
19	Nafas	0.5	0.64	0.33	0.5	0.5	0	0.5	0.75	0.46
20	Tuhoon	0.44	0.68	0.44	1	0.5	0.14	0.5	0.75	0.52
21	Kun Being	0.38	0.61	0.44	1	0.5	0.14	0	0.38	0.44
22	Tawazon	0.5	0.68	0.33	1	0.5	0	0.5	0.75	0.49
Australian apps										
	HeadGear	0.88	0.93	0.78	1	1	0.57	0.5	0.75	0.81
	ReachOutWorryTime	0.75	0.86	0.67	1	1	0.71	0	1	0.77
	Smiling Mood	0.88	0.86	0.78	1	1	0.29	0.5	1	0.77
	MoodMission	0.75	0.79	0.44	1	1	0.57	0.5	0.75	0.69

Arabic mHealth apps scored the lowest in the safety, technical support and update, and security subscales, with 15 (app 4–11,13–16,18,19,22) obtaining a score of 0 in the safety subscale. For technical support, ten apps (app 2,7,8,10,13–17,21) scored zero and 12 apps (app 1,3–6, 9,11,12,18–20,22) received a moderate score of 0.5, indicating a lack of customer support on those apps. Most (15/22) apps scored below a moderate level of security, with three (app 13,14,16) scoring 0. However, Arabic apps performed well on technology, including app functionality, function retrieval after context changes, resource efficiency, and data recovery, with nearly all (21/22) apps scoring 0.75.

On the privacy subscale, 13 Arabic apps scored 0.5 and above, while three apps (app 13,14,16) scored zero, indicating that no privacy safeguards exist, and six apps scored below the moderate level. Indeed, four apps had no privacy policies and lacked important information. Few apps guaranteed the privacy of recorded information. No apps asked permission for data to be used by third parties and/or commercial parties, and none state that data would be erased when the service is finished. Apps scored higher on usability subscales than privacy, with the majority (18/22) scoring 0.5 or above. However, before publication, none were co-designed or assessed by potential users to ensure usability and adaptability.

Scores for appropriateness and suitability, including explaining the benefits and advantages of using the app to user and the inclusion of expert input in app development, show significant patterns. Notably, only four Arabic apps (app 17,20–22) obtained a perfect appropriateness score. In comparison, six apps (app 7,9,10,14–16) received an appropriateness score of zero, indicating a lack of input from specialized individuals or health organizations, and the other 12 apps received a moderate appropriateness score of 0.5 given that these apps inform users about the benefits and advantages of using the app, yet lack expert input. Similarly, in transparency and content subscales, which concern the use of scientific evidence to ensure app quality and compliance with ethical principles and values, three apps (app 1,2,3) obtained a perfect appropriateness score of one. Four apps (app 13,15,16,18) received an appropriateness score of zero.

Considering the Australian apps, HeadGear received the highest overall MAG score (0.81), followed by Smiling Mood (0.77), ReachOutWorryTime (0.77), and MoodMission (0.69) (see Table 3). Australian apps scored well on appropriateness, suitability, transparency, and content. All apps scored perfectly for professional involvement, scientific evidence, and ethical standards reflecting high-quality content. Australian apps also ranked well in technology, usability, and privacy subscales. Regarding technology, two apps (Smiling Mood, ReachOutWorryTime) received a perfect score of one, while the remaining two (HeadGear, MoodMission) received a score of 0.75. These apps are technically well-developed, ensuring seamless functionality and efficient resource usage, easily handling context changes and external interruptions. All apps performed well on privacy subscales, receiving a score of 0.79 or higher. They also demonstrated good usability, with all receiving scores of 0.75 or higher, indicating user-friendly design and efficient navigation. For security, three apps received moderately high scores (ranging from 0.67 to 0.78), and one received a score of 0.44. Although Australian apps have a higher level of security than Arabic apps, there may be opportunities for improvement to ensure better data protection and general reliability by adding even more robust safety measures. In general, Australian apps fail to provide information and clear terms and conditions for cloud services, including necessary security measures, and there is a lack of clarity regarding the use of encryption mechanisms for data storage, collection, and exchange. Similarly, in the safety subscales, three apps (HeadGear, MoodMission, ReachOutWorryTime) received moderate to moderately high scores (ranging from 0.57 to 0.71), while one (Smiling Mood) received a score of 0.29. There was a significant gap in providing users with information concerning potential hazards caused by incorrect app usage. Apps lacked a way to notify users of emerging problems with use, thus allowing them to delete the app and avoid potential risks. One app (ReachOutWorryTime) failed the technical support subscales, while the other three (HeadGear, MoodMission, Smiling Mood) received a moderate score of 0.5.

## 4 Discussion

Our systematic review of mental mHealth apps available from Arabic iOS and Android stores identified 22 apps for Arabic speakers, representing less than 1% of the mHealth apps searched. In comparison, between 10,000 and 20,000 mental mHealth apps are available for download in the Western market (41, 42). We found that Arabic mental mHealth apps primarily offer online counselling services, enabling remote treatment delivery options, allowing real-time communication with specialists via video, text, or audio calls, but lack evidence-based information and functionalities for self-directed support. This focus reflects the limited availability of face-to-face consultations (4), but could also be driven by market demand for alternative private mental health services, where individuals can seek treatment anonymously to avoid stigma and judgment. Noorwali et al. reported that online therapy promotes mental health help-seeking among young individuals in Saudi Arabia by providing efficient outcomes, anonymity, stigma avoidance, and accessibility when compared with in-person sessions (43).

Arabic apps scored highest in functionality and aesthetics on MARS subscales but appeared low in subjective quality, engagement, and information scores, indicating a specific intervention design gap. In general, Arabic apps tend to be logically structured and run smoothly with no crashes or significant lag, with a consistent visual design that aligns with the app's overall theme, using appropriate layout, color, and size for buttons, icons, and content. On the MAG scale, most Arabic apps had low scores for safety, security, privacy, usability, appropriateness and suitability, transparency and content, technical support and update, with safety receiving the lowest ratings.

Only two apps (apps 15,16) use interventions that emphasized psychoeducation, but these received poor design scores based on MAG and MARS scales, suggesting a failure to satisfy the minimum standards and requirements of safety, security, evidence-based psychoeducation and a failure to deliver information in a culturally sensitive manner. Arabic apps generally offered insufficient information quality and quantity, with minimal visual representation, confirming a previous report (44). They missed funding from, support by, or affiliation with healthcare providers, authorities, or other nationally recognized research organizations, suggesting that the market lacks evidence-based products and is not well integrated with digital health research, which is in its early stages in Arabic countries (45).

Particularly for Arabic people culturally appropriate psychoeducation is likely a critical consideration for mental mHealth apps, given low public awareness of mental illness and associated stigma are associated with poor treatment engagement across age groups (13, 43). Employing culturally specific metaphors, proverbs, stories, and analogies to appropriately convey information could help raise awareness and reduce stigma around mental disorders (46, 47).

Importantly, our analysis reveals that in contrast to Australian apps, Arabic apps lack evidence-based interventions such as CBT, self-help tools for behaviour change, skill acquisition, cognitive restructuring, and mood/emotion tracking. We found evidence of some Arabic apps that provided guided stress and anxiety relief through breathing, muscle relaxation, and meditation exercises. Evidence to support the effectiveness of app-guided mindfulness is growing (48). Muscle relaxation and breathing exercises are part of many religious and spiritual traditions and are widely practised in non-Western cultures (47). However, one study has suggested that the Islamic worldview was associated with poor responses to app-based mindfulness meditation (49), suggesting that cultural characteristics and religious beliefs may influence meditation experiences and benefits. Additionally, Arabic mindfulness/meditation apps investigated in our study provided no information on potential clinical benefits to inform user engagement. Further research is needed to investigate potential cultural factors influencing Arabs' use of mindfulness/meditation apps and their efficacy. The lack of evidence-based self-help strategies and techniques in Arabic apps significantly impacts Arabic apps' quality.

Interventions for mental illness must be tailored to the specific cultural context to help adapt and translate evidence-based interventions, resulting in more effective treatment implementation and better outcomes for Arab patients (13). Religion is a cultural identity marker, and Islam remains the most dominant religion and element in shaping Arabic culture (50, 51). In faith-based Arab societies, many individuals may not value Western evidence-based psychological interventions. Religious beliefs and practices strongly affect how Arabs perceive mental illness, affect their preferences for mental health care, and affect their willingness to seek professional help when necessary (52). Arabs prefer consulting religious leaders or receiving treatment from religious healers before or during mental health treatment since this method is viewed as less stigmatizing and is widely accepted within the society (53, 54). Indeed, two apps in our review (app 14,20) employed Islamic-based content (e.g., supplication) and guided Islamic teaching-inspired meditation to help alleviate mental health disorders. As discussed, recent research aims to integrate religion with evidence-based intervention such as CBT (55–57). Religion-adapted cognitive behavioural therapy (R-CBT) incorporates patients' religious beliefs into secular CBT procedures (55). R-CBT has been shown to improve the purpose in life (PIL) (58), and strengthen the therapeutic alliance (59) in depressed religious patients, compared with standard CBT. Several techniques to make R-CBT interventions more relatable and effective for religious clients, including cognitive restructuring with religious content, religious imagery modification, behavioural activation through religious activities, psychoeducation using religious theories, contemplative prayer and meditation, and motivational strategies using religious content (55).

Indeed, low adoption and acceptance of digital interventions may occur due to a mismatch between user's personal needs and preferences, digital skills, and context (60). Aryana and Brewster, argued that, in general, mental mHealth apps' low proof of efficacy has been exacerbated by a lack of design methodologies because developers frequently regard mental health apps as standalone products and dismiss the complicated context of use (61). The importance of collaboration/participatory development “with” stakeholders rather than “for” them when developing digital interventions tailored to user's needs and context is emphasized by van Gemert-Pijnen et al. One approach to participatory development is co-design, ensuring developed tools and interventions are more relevant to the target population's (end-user) demands, along with identifying potential barriers to acceptance and use (62, 63). Research on co-design approaches for enhancing mental health programs/services for culturally and linguistically diverse groups is limited (64). Culturally tailored interventions involving the target group/s in the co-design can be useful in identifying any exceptional conditions or requirements that may be peculiar to those groups (65). Given this, co-designing mental health apps for Arab users could create more effective and user-friendly interventions.

Guidelines for incorporating mHealth technologies into mental health care recommend that mHealth apps are (1) grounded in scientific evidence and incorporate established therapeutic techniques; (2) thoughtfully designed, taking into account user experience, interface design, and usability to improve engagement and effectiveness; (3) prioritize quality and privacy regulations for user safety and data protection; (4) provide immediate psychological support and facilitate self-monitoring of mood, behaviour, or symptoms, to give users valuable insights and feedback that supports their mental health; and (5) promote healthy behaviour changes and increased adherence to treatment programmes (23, 66). Also recommended is to regularly maintain, modify, and improve apps to reflect advances in technology and psychological research, addressing technical issues that may arise as hardware and software systems change (23). In our review, the Arabic apps reviewed are of poor quality with limited functionality, making them ineffective in meeting demand and showing major gaps in usability, engagement, information quality, safety, privacy, security, and scientific evidence. While a recent study showed relatively high uptake of mHealth app usage among Jordanian outpatients (67) finding that 41.6% of the 438 outpatients surveyed had downloaded mHealth apps, over two-thirds of participants (70%) stopped using the apps they downloaded, suggesting a lack of long term perceived benefit. Lack of trust and complexity of the apps were among the reasons reported for never downloading mHealth app (67).

Arabic apps in this study lack the features and qualities necessary for an engaging experience and, unlike Australian apps, do not prioritize building individualized user experiences to facilitate behavioural change, cognitive restructuring, using mood/emotion tracking features. These findings align with (68), who suggested that Arabic mental well-being apps displayed poorly implemented engagement features and essential functions such as sharing and reminders. Indeed, mental health apps could fail to engage users, particularly in cases of depression and schizophrenia, when motivation is often low (69). Engagement with mental mHealth apps helps effective interventions or therapies in real-life contexts. Therefore, it is vital to keep the user in mind throughout the app design and development.

Research suggests that existing mental mHealth apps have not generally been fully evaluated, raising concerns about their safety, efficacy, and impact (70, 71). For instance, a recent systematic review (72) of the top 100 mobile apps for bipolar disorder reveals insufficient academic research about the effectiveness of the apps accessible in the marketplace, with only one app supported by a peer-reviewed study. Similarly, a systematic review of 293 apps found that only 3.41% had published studies on their effectiveness (73). Consistent with this evidence, the Arabic apps reviewed did not provide proof of end-user evaluation prior to release. They scored below moderate to moderate on the usability subscale on MAG, which may dissuade users from incorporating apps into their daily routines, resulting in decreased usage or rejection.

Our analysis shows that Arabic apps place a low priority on reliable and safe patient-centred intervention and do not take proper measures and include mechanisms to help users make informed decisions to help them avoid potential harm. Indeed, our descriptive analysis shows that the majority of the Arabic apps, which are primarily counselling apps, rely on the counsellor/clinician being in contact with the patient to implement a risk management plan; only one app gives the user an option to add a phone number of a relative or friend to contact in case of emergency. Similarly, in most Arabic apps, users are not encouraged to contact hotlines or mental health specialists in emergencies. Only two apps provided emergency service phone numbers, and three offered contact numbers for domestic abuse/violence support, while only two counselling apps offered urgent online counselling (Supplementary Tables S10). Martinengo et al. found that over 2 million mental mHealth apps they reviewed had either inaccurate crisis helpline information or none, suggesting this is part of a wider problem (74).

Mental mHealth apps collect sensitive data, raising concerns about privacy and security because most apps are not subject to healthcare privacy legislation, especially concerning when this data is actively marketed (69). Prioritizing data protection and user privacy in mental mHealth apps is vital to protect privacy because these qualities can affect user engagement and trust. Most Arabic mental mHealth apps in this study generally lacked privacy measures to protect users' health information and lacked transparency in data storage, access, and handling. Critically, all mental mHealth apps should provide clear and detailed information about collected data, the rationale for such collection, commercial data sharing practices, data encryption and storage, access rights, and user controls.

Based on our study, we believe the quality and effectiveness of mental mHealth for the Arab consumer can be improved. Usability, usefulness, infrastructure, data security, privacy, and safety are concerns for currently available apps. To answer these concerns, along with culturally appropriateness and support for mental health emergencies, and to expand adoption, it is critical to develop apps using an evidence-based CBT framework. Improved quality and uptake may lead to more investment in Arabic digital mental health care, a possible lucrative market, given the size of the population and the service gap. Further quantitative and qualitative research is needed to develop effective and engaging mental mHealth for Arab users. Frameworks to help and guide developers to “best practice” on designing mHealth tools that deliver an appropriate standard of mental health care for Arabs are needed. Involving end-users and specialized professionals in the design process could help to improve the quality, reliability, and efficacy of Arabic mental mHealth apps. Stakeholders (e.g., universities, governments, technology companies, investors, suppliers, and strategists) could accelerate the realization and adoption of mental mHealth in Arab countries.

## 5 Strengths and limitations of the study

The strengths of this review include the implementation of a stringent multistep methodology for app evaluation using both the MARS and MAG rating scales. To our knowledge, this is the first study to utilize both the MARS and MAG frameworks for a comprehensive assessment of mental mHealth apps. Adding to the work by Alhuwail et al. (44), we performed a descriptive comparative analysis of the Arabic applications compared to Australian ones to better understand the strengths and weaknesses of the Arabic apps' features and quality. Limitations of this systematic review were two-fold: examining only freely available apps, excluding paid extensions; existing mHealth apps are frequently updated, and new ones are introduced constantly.

## Data Availability

The original contributions presented in the study are included in the article/Supplementary Material, further inquiries can be directed to the corresponding author.

## Footnotes

^1^ https://asotools.io/apps
