# Supplementary material for: A systematic review of features and content quality of Arabic mental mHealth apps
Source: Front Digit Health. 2024 Dec 11;6:1472251. doi: 10.3389/fdgth.2024.1472251 (PMC11668747; doi:10.3389/fdgth.2024.1472251)
Supplement: Supplementary file 1 [file Table1.docx]

Supplementary Material

# Supplementary Tables

**Supplemental Table S1:** Inclusion of professional support features.

| **No.** | **App** | **Professional support features** | | | | | | | |
| --- | --- | --- | --- | --- | --- | --- | --- | --- | --- |
|  |  | Video-based support | Audio-based support | Text-based support | Offline consultation booking | Bookings history record | Rating system | Reviews & feedback system | Like functionality |
| **1** | **Estenarh** | **YES** | **YES** | **YES** |  | **YES** | **YES** | **YES** |  |
| **2** | **Shezlong** | **YES** | **YES** | **YES** |  | **YES** | **YES** | **YES** |  |
| **3** | **O7 Therapy** | **YES** | **YES** | **YES** |  | **YES** |  |  |  |
| **4** | **Labayh** | **YES** | **YES** | **YES** |  | **YES** | **YES** | **YES** |  |
| **5** | **Ayadi** | **YES** |  | **YES** |  | **YES** |  | **YES** |  |
| **6** | **Tetaman** | **YES** | **YES** | **YES** |  | **YES** | **YES** | **YES** | **YES** |
| **7** | **AlMorshed** | **YES** | **YES** | **YES** | **YES** |  | **YES** |  |  |
| **8** | **Mind** | **YES** | **YES** | **YES** |  | **YES** | **YES** | **YES** |  |
| **9** | **Faserly** | **YES** | **YES** | **YES** |  |  | **YES** | **YES** |  |
| **10** | **Daeim** | **YES** |  |  |  | **YES** | **YES** | **YES** |  |
| **11** | **Cura** | **YES** | **YES** | **YES** |  | **YES** | **YES** | **YES** |  |
| **12** | **Sanar** | **YES** |  |  |  | **YES** |  |  |  |
| **13** | **Akhtibar Alshakhsia Alnarjisia** |  |  |  |  |  |  |  |  |
| **14** | **Alruqayat Alshareia** |  |  |  |  |  |  |  |  |
| **15** | **Alsiha Alnafsia** |  |  |  |  |  |  |  |  |
| **16** | **Alsiha Alnafsia w aleaqlia** |  |  |  |  |  |  |  |  |
| **17** | **Daliluk Alnafsi** |  |  |  |  |  |  |  |  |
| **18** | **Tawkidat** |  |  |  |  |  |  |  |  |
| **19** | **Nafas** |  |  |  |  |  |  |  |  |
| **20** | **Tuhoon** |  |  |  |  |  |  |  |  |
| **21** | **Kun Being** |  |  |  |  |  |  |  |  |
| **22** | **Tawazon** |  |  |  |  |  |  |  |  |
| **-** | **Smiling Mind** |  |  |  |  |  |  |  |  |
| **-** | **ReachOut WorryTime** |  |  |  |  |  |  |  |  |
| **-** | **HeadGear** |  |  |  |  |  |  |  |  |
| **-** | **MoodMission** |  |  |  |  |  |  |  |  |

**Supplemental Table S2:** Inclusion of personalized/customized features.

| **No.** | **App** | **Personalized/customized experience features** | | | | |
| --- | --- | --- | --- | --- | --- | --- |
|  |  | Tailored search filters (e.g. According cost,gender,etc) | Background color customization | Background sound customization | App's icon color customization | Set display preference (e.g. Light mode, dark mode) |
| **1** | **Estenarh** | **YES** |  |  |  |  |
| **2** | **Shezlong** | **YES** |  |  |  |  |
| **3** | **O7 Therapy** | **YES** |  |  |  |  |
| **4** | **Labayh** | **YES** |  |  |  |  |
| **5** | **Ayadi** | **YES** |  |  |  |  |
| **6** | **Tetaman** | **YES** |  |  |  |  |
| **7** | **AlMorshed** |  |  |  |  |  |
| **8** | **Mind** | **YES** |  |  |  |  |
| **9** | **Faserly** | **YES** |  |  |  |  |
| **10** | **Daeim** | **YES** |  |  |  |  |
| **11** | **Cura** | **YES** |  |  |  |  |
| **12** | **Sanar** | **YES** |  |  |  |  |
| **13** | **Akhtibar Alshakhsia Alnarjisia** |  |  |  |  |  |
| **14** | **Alruqayat Alshareia** |  |  |  |  |  |
| **15** | **Alsiha Alnafsia** |  |  |  |  |  |
| **16** | **Alsiha Alnafsia w aleaqlia** |  |  |  |  |  |
| **17** | **Daliluk Alnafsi** | **YES** |  |  |  |  |
| **18** | **Tawkidat** |  | **YES** |  | **YES** |  |
| **19** | **Nafas** |  |  |  |  |  |
| **20** | **Tuhoon** |  |  |  |  |  |
| **21** | **Kun Being** |  |  |  |  |  |
| **22** | **Tawazon** |  |  | **YES** |  |  |
| **-** | **Smiling Mind** |  |  | **YES** |  | **YES** |
| **-** | **ReachOut WorryTime** |  |  |  |  |  |
| **-** | **HeadGear** |  |  |  |  |  |
| **-** | **MoodMission** |  |  |  |  |  |

**Supplemental Table S3**: Inclusion of mental health education and self-awareness features.

| **No.** | **App** | **Mental health education and self-awareness features** | | | | | | | | | | |
| --- | --- | --- | --- | --- | --- | --- | --- | --- | --- | --- | --- | --- |
|  |  | Text-based information | Visual information (charts, graphs, images, video) | Podcast | Blog | Web links | Online webinars/ workshops/ courses | Favorites functionality | Information/ materials sharing functionality | Self-report assessment of the risk/severity of a mental condition | Log of self-assessment scores | Mental health care and support service search |
| **1** | **Estenarh** | **YES** |  |  |  |  |  |  |  |  |  |  |
| **2** | **Shezlong** | **YES** |  |  | **YES** |  |  |  |  | **YES** |  |  |
| **3** | **O7 Therapy** | **YES** | **YES** |  |  |  | **YES (with fee)** |  |  |  |  |  |
| **4** | **Labayh** | **YES** | **YES** |  | **YES** |  | **YES (with fee)** | **YES** | **YES** | **YES** | **YES** |  |
| **5** | **Ayadi** | **YES** |  |  |  |  |  |  | **YES** |  |  |  |
| **6** | **Tetaman** | **YES** |  |  |  |  |  |  | **YES** |  |  |  |
| **7** | **AlMorshed** | **YES** |  |  | **YES** |  |  |  | **YES** | **YES** |  |  |
| **8** | **Mind** | **YES** |  |  | **YES** |  | **YES** |  |  | **YES (with fee)** |  |  |
| **9** | **Faserly** | **YES** | **YES** |  | **YES** |  |  |  | **YES** |  |  |  |
| **10** | **Daeim** | **YES** |  |  |  | **YES** |  |  |  |  |  |  |
| **11** | **Cura** | **YES** |  |  |  |  |  |  | **YES** |  |  |  |
| **12** | **Sanar** | **YES** |  |  |  |  |  |  | **YES** |  |  |  |
| **13** | **Akhtibar Alshakhsia Alnarjisia** |  |  |  |  |  |  |  |  | **YES** |  |  |
| **14** | **Alruqayat Alshareia** |  |  |  |  |  |  |  |  |  |  |  |
| **15** | **Alsiha Alnafsia** | **YES** |  |  |  |  |  | **YES** | **YES** |  |  |  |
| **16** | **Alsiha Alnafsia w aleaqlia** | **YES** |  |  |  |  |  |  |  |  |  |  |
| **17** | **Daliluk Alnafsi** | **YES** |  |  |  | **YES** |  |  | **YES** |  |  | **YES** |
| **18** | **Tawkidat** |  |  |  |  |  |  |  |  |  |  |  |
| **19** | **Nafas** |  |  | **YES (subscription)** |  |  |  | **YES** | **YES** |  |  |  |
| **20** | **Tuhoon** |  |  | **YES** |  |  |  | **YES** | **YES** |  |  |  |
| **21** | **Kun Being** |  |  |  |  |  |  |  |  |  |  |  |
| **22** | **Tawazon** |  |  | **YES** |  |  |  | **YES** | **YES** |  |  |  |
| **-** | **Smiling Mind** | **YES** | **YES** |  |  | **YES** |  |  |  |  |  |  |
| **-** | **ReachOut WorryTime** |  |  |  |  |  |  |  |  |  |  |  |
| **-** | **HeadGear** | **YES** | **YES** |  |  |  |  |  |  | **YES** |  |  |
| **-** | **MoodMission** | **YES** | **YES** |  |  |  |  |  |  | **YES** |  |  |

**Supplemental Table S4:** Inclusion of social networks and support features.

| **No.** | **App** | **Social networks and support features** | | | |
| --- | --- | --- | --- | --- | --- |
|  |  | Group therapy | App community | Progress/self-assessment outcome sharing (Facebook, Twitter, WhatsApp, etc) |  |
| **1** | **Estenarh** |  |  |  |  |
| **2** | **Shezlong** |  |  |  |  |
| **3** | **O7 Therapy** | **YES** |  |  |  |
| **4** | **Labayh** | **YES** | **YES** | **YES** |  |
| **5** | **Ayadi** |  |  |  |  |
| **6** | **Tetaman** |  |  |  |  |
| **7** | **AlMorshed** |  |  |  |  |
| **8** | **Mind** |  |  |  |  |
| **9** | **Faserly** |  |  |  |  |
| **10** | **Daeim** |  |  |  |  |
| **11** | **Cura** |  |  |  |  |
| **12** | **Sanar** |  |  |  |  |
| **13** | **Akhtibar Alshakhsia Alnarjisia** |  |  | **YES** |  |
| **14** | **Alruqayat Alshareia** |  |  |  |  |
| **15** | **Alsiha Alnafsia** |  |  |  |  |
| **16** | **Alsiha Alnafsia w aleaqlia** |  |  |  |  |
| **17** | **Daliluk Alnafsi** |  |  |  |  |
| **18** | **Tawkidat** |  |  |  |  |
| **19** | **Nafas** |  |  |  |  |
| **20** | **Tuhoon** |  |  |  |  |
| **21** | **Kun Being** |  |  |  |  |
| **22** | **Tawazon** |  |  |  |  |
| **-** | **Smiling Mind** |  |  |  |  |
| **-** | **ReachOut WorryTime** |  |  |  |  |
| **-** | **HeadGear** |  |  |  |  |
| **-** | **MoodMission** |  |  |  |  |

**Supplemental Table S5:** Inclusion of mindfulness/ meditation/ relaxation features.

| **No.** | **App** | **Mindfulness/ meditation/ relaxation features** | | | | | | | | | | | | |
| --- | --- | --- | --- | --- | --- | --- | --- | --- | --- | --- | --- | --- | --- | --- |
|  |  | One-guided-audio-session | Guided-audio-module (consist of number of sessions laid out in stages) | Music and natural sounds | Audio-based Recitation (Ruqyah) | Audio-based supplication (Duaa) and remembrance (Thiker) | Guided Islamic teaching-inspired meditation | Audio book summaries | Web links | Breathing exercises | Offline downloads | Meditation sharing functionality | Favorites functionality | Meditation stats |
| **1** | **Estenarh** |  |  |  |  |  |  |  |  |  |  |  |  |  |
| **2** | **Shezlong** |  |  |  |  |  |  |  |  |  |  |  |  |  |
| **3** | **O7 Therapy** |  |  |  |  |  |  |  |  |  |  |  |  |  |
| **4** | **Labayh** |  |  |  |  |  |  |  |  |  |  |  |  |  |
| **5** | **Ayadi** |  |  |  |  |  |  |  |  |  |  |  |  |  |
| **6** | **Tetaman** |  |  |  |  |  |  |  |  |  |  |  |  |  |
| **7** | **AlMorshed** |  |  |  |  |  |  |  |  |  |  |  |  |  |
| **8** | **Mind** |  |  |  |  |  |  |  |  |  |  |  |  |  |
| **9** | **Faserly** |  |  |  |  |  |  |  |  |  |  |  |  |  |
| **10** | **Daeim** |  |  |  |  |  |  |  |  |  |  |  |  |  |
| **11** | **Cura** |  |  |  |  |  |  |  |  |  |  |  |  |  |
| **12** | **Sanar** |  |  |  |  |  |  |  |  |  |  |  |  |  |
| **13** | **Akhtibar Alshakhsia Alnarjisia** |  |  |  |  |  |  |  |  |  |  |  |  |  |
| **14** | **Alruqayat Alshareia** |  |  |  | **YES** | **YES** |  |  | **YES** |  | **YES** |  |  |  |
| **15** | **Alsiha Alnafsia** |  |  |  |  |  |  |  |  |  |  |  |  |  |
| **16** | **Alsiha Alnafsia w aleaqlia** |  |  |  |  |  |  |  |  |  |  |  |  |  |
| **17** | **Daliluk Alnafsi** |  |  |  |  |  |  |  |  |  |  |  |  |  |
| **18** | **Tawkidat** |  |  |  |  |  |  |  |  |  |  |  |  |  |
| **19** | **Nafas** | **YES** | **YES** | **YES** |  |  |  |  |  | **YES** |  | **YES** | **YES** | **YES** |
| **20** | **Tuhoon** | **YES** | **YES** | **YES** |  |  | **YES** | **YES** |  | **YES** | **YES** | **YES** | **YES** | **YES** |
| **21** | **Kun Being** | **YES** | **YES** |  |  |  |  |  |  | **YES** | **YES** | **YES** | **YES** | **YES** |
| **22** | **Tawazon** | **YES** | **YES** | **YES** |  |  |  |  |  | **YES** | **YES** | **YES** | **YES** | **YES** |
| **-** | **Smiling Mind** | **YES** | **YES** |  |  |  |  |  |  | **YES** | **YES** | **YES** | **YES** | **YES** |
| **-** | **ReachOut WorryTime** |  |  |  |  |  |  |  |  |  |  |  |  |  |
| **-** | **HeadGear** | **YES** |  |  |  |  |  |  |  | **YES** |  |  |  |  |
| **-** | **MoodMission** |  |  |  |  |  |  |  |  |  |  |  |  |  |

**Supplemental Table S6:** Inclusion of behavioural activation/ skill acquisition/ coping features.

| **No.** | **App** | **Behavioural activation/ skill acquisition/ coping features** | | | | | | | |
| --- | --- | --- | --- | --- | --- | --- | --- | --- | --- |
|  |  | Challenges/missions | Correlate challenges/missions with reported mood state | Functional analysis of mission/challenge (i.e. Justify the mission/challenge) | Alternatives/options for missions/ activities/exercises | Request user to record completed mission/tasks/ activities ( track completed tasks/ activities ) | Post-mission reflection (questions) | Favourites mission/task/activity | Information and statistics about user progress and completed tasks |
| **1** | **Estenarh** |  |  |  |  |  |  |  |  |
| **2** | **Shezlong** |  |  |  |  |  |  |  |  |
| **3** | **O7 Therapy** |  |  |  |  |  |  |  |  |
| **4** | **Labayh** |  |  |  |  |  |  |  |  |
| **5** | **Ayadi** |  |  |  |  |  |  |  |  |
| **6** | **Tetaman** |  |  |  |  |  |  |  |  |
| **7** | **AlMorshed** |  |  |  |  |  |  |  |  |
| **8** | **Mind** |  |  |  |  |  |  |  |  |
| **9** | **Faserly** |  |  |  |  |  |  |  |  |
| **10** | **Daeim** |  |  |  |  |  |  |  |  |
| **11** | **Cura** |  |  |  |  |  |  |  |  |
| **12** | **Sanar** |  |  |  |  |  |  |  |  |
| **13** | **Akhtibar Alshakhsia Alnarjisia** |  |  |  |  |  |  |  |  |
| **14** | **Alruqayat Alshareia** |  |  |  |  |  |  |  |  |
| **15** | **Alsiha Alnafsia** |  |  |  |  |  |  |  |  |
| **16** | **Alsiha Alnafsia w aleaqlia** |  |  |  |  |  |  |  |  |
| **17** | **Daliluk Alnafsi** |  |  |  |  |  |  |  |  |
| **18** | **Tawkidat** |  |  |  |  |  |  |  |  |
| **19** | **Nafas** |  |  |  |  |  |  |  |  |
| **20** | **Tuhoon** |  |  |  |  |  |  |  |  |
| **21** | **Kun Being** |  |  |  |  |  |  |  |  |
| **22** | **Tawazon** |  |  |  |  |  |  |  |  |
| **-** | **Smiling Mind** |  |  |  |  |  |  |  |  |
| **-** | **ReachOut WorryTime** |  |  |  |  |  |  |  |  |
| **-** | **HeadGear** | **YES** |  | **YES** |  | **YES** |  |  | **YES** |
| **-** | **MoodMission** | **YES** | **YES** | **YES** | **YES** | **YES** | **YES** | **YES** | **YES** |

**Supplemental Table S7:** Inclusion of cognitive restructuring features.

| **No.** | **App** | **Cognitive restructuring features** | | | | | | | | | | |
| --- | --- | --- | --- | --- | --- | --- | --- | --- | --- | --- | --- | --- |
|  |  | Journaling | Worry relive diary | Timed worry reflections | Worry time duration length setting | Ditch of recorded worry thoughts that no longer matter | Worry thought count | Pre-built affirmations | Favourite affirmations | Personal affirmations crafting | Affirmation sharing functionality | Affirmation timer setting |
| **1** | **Estenarh** |  |  |  |  |  |  |  |  |  |  |  |
| **2** | **Shezlong** |  |  |  |  |  |  |  |  |  |  |  |
| **3** | **O7 Therapy** |  |  |  |  |  |  |  |  |  |  |  |
| **4** | **Labayh** |  |  |  |  |  |  |  |  |  |  |  |
| **5** | **Ayadi** |  |  |  |  |  |  |  |  |  |  |  |
| **6** | **Tetaman** |  |  |  |  |  |  |  |  |  |  |  |
| **7** | **AlMorshed** |  |  |  |  |  |  |  |  |  |  |  |
| **8** | **Mind** |  |  |  |  |  |  |  |  |  |  |  |
| **9** | **Faserly** |  |  |  |  |  |  |  |  |  |  |  |
| **10** | **Daeim** |  |  |  |  |  |  |  |  |  |  |  |
| **11** | **Cura** |  |  |  |  |  |  |  |  |  |  |  |
| **12** | **Sanar** |  |  |  |  |  |  |  |  |  |  |  |
| **13** | **Akhtibar Alshakhsia Alnarjisia** |  |  |  |  |  |  |  |  |  |  |  |
| **14** | **Alruqayat Alshareia** |  |  |  |  |  |  |  |  |  |  |  |
| **15** | **Alsiha Alnafsia** |  |  |  |  |  |  |  |  |  |  |  |
| **16** | **Alsiha Alnafsia w aleaqlia** |  |  |  |  |  |  |  |  |  |  |  |
| **17** | **Daliluk Alnafsi** |  |  |  |  |  |  |  |  |  |  |  |
| **18** | **Tawkidat** |  |  |  |  |  |  | **YES** | **YES** | **YES** | **YES** | **YES** |
| **19** | **Nafas** |  |  |  |  |  |  |  |  |  |  |  |
| **20** | **Tuhoon** | **YES (subscription)** |  |  |  |  |  |  |  |  |  |  |
| **21** | **Kun Being** |  |  |  |  |  |  |  |  |  |  |  |
| **22** | **Tawazon** |  |  |  |  |  |  |  |  |  |  |  |
| **-** | **Smiling Mind** |  |  |  |  |  |  |  |  |  |  |  |
| **-** | **ReachOut WorryTime** |  | **YES** | **YES** | **YES** | **YES** | **YES** |  |  |  |  |  |
| **-** | **HeadGear** |  | **YES** | **YES** |  | **YES** |  |  |  |  |  |  |
| **-** | **MoodMission** |  |  |  |  |  |  |  |  |  |  |  |

**Supplemental Table S8:** Inclusion of mood / emotions tracking features.

| **No.** | **App** | **Mood / Emotions tracking features** | | | |
| --- | --- | --- | --- | --- | --- |
|  |  | Enquire about the user's current state of mood/emotions. | Questionnaire that assesses the state of mood/emotions ( track mood/emotions) | Graph of mood/emotion tracking |  |
| **1** | **Estenarh** |  |  |  |  |
| **2** | **Shezlong** |  |  |  |  |
| **3** | **O7 Therapy** |  |  |  |  |
| **4** | **Labayh** | **YES** |  | **YES** |  |
| **5** | **Ayadi** |  |  |  |  |
| **6** | **Tetaman** |  |  |  |  |
| **7** | **AlMorshed** |  |  |  |  |
| **8** | **Mind** |  |  |  |  |
| **9** | **Faserly** |  |  |  |  |
| **10** | **Daeim** |  |  |  |  |
| **11** | **Cura** |  |  |  |  |
| **12** | **Sanar** |  |  |  |  |
| **13** | **Akhtibar Alshakhsia Alnarjisia** |  |  |  |  |
| **14** | **Alruqayat Alshareia** |  |  |  |  |
| **15** | **Alsiha Alnafsia** |  |  |  |  |
| **16** | **Alsiha Alnafsia w aleaqlia** |  |  |  |  |
| **17** | **Daliluk Alnafsi** |  |  |  |  |
| **18** | **Tawkidat** |  |  |  |  |
| **19** | **Nafas** |  |  |  |  |
| **20** | **Tuhoon** | **YES** |  | **YES** |  |
| **21** | **Kun Being** |  |  |  |  |
| **22** | **Tawazon** | **YES** |  | **YES** |  |
| **-** | **Smiling Mind** |  | **YES** |  |  |
| **-** | **ReachOut WorryTime** |  |  |  |  |
| **-** | **HeadGear** | **YES** |  | **YES** |  |
| **-** | **MoodMission** |  | **YES** |  |  |

**Supplemental Table S9:** Inclusion of therapeutic features.

| **No.** | **App** | **Features enhance therapeutic commitment** | | |
| --- | --- | --- | --- | --- |
|  |  | Reminders and alerts | Gamification strategy and rewards (e.g.level upgrades, points, medals) | Congratulatory and encouraging messages/feedback |
| **1** | **Estenarh** | **YES** |  |  |
| **2** | **Shezlong** | **YES** |  |  |
| **3** | **O7 Therapy** | **YES** |  |  |
| **4** | **Labayh** | **YES** |  |  |
| **5** | **Ayadi** | **YES** |  |  |
| **6** | **Tetaman** | **YES** |  |  |
| **7** | **AlMorshed** |  |  |  |
| **8** | **Mind** | **YES** |  |  |
| **9** | **Faserly** | **YES** |  |  |
| **10** | **Daeim** | **YES** |  |  |
| **11** | **Cura** | **YES** |  |  |
| **12** | **Sanar** | **YES** |  |  |
| **13** | **Akhtibar Alshakhsia Alnarjisia** |  |  |  |
| **14** | **Alruqayat Alshareia** |  |  |  |
| **15** | **Alsiha Alnafsia** |  |  |  |
| **16** | **Alsiha Alnafsia w aleaqlia** |  |  |  |
| **17** | **Daliluk Alnafsi** |  |  |  |
| **18** | **Tawkidat** | **YES** |  |  |
| **19** | **Nafas** | **YES** |  |  |
| **20** | **Tuhoon** | **YES** | **YES** | **YES** |
| **21** | **Kun Being** | **YES** |  |  |
| **22** | **Tawazon** | **YES** |  |  |
| **-** | **Smiling Mind** | **YES** |  |  |
| **-** | **ReachOut WorryTime** | **YES** |  |  |
| **-** | **HeadGear** | **YES** | **YES** |  |
| **-** | **MoodMission** | **YES** | **YES** | **YES** |

**Supplemental Table S10:** Inclusion of crisis intervention and aid features.

| **No.** | **App** | **Crisis intervention and aid features** | | |
| --- | --- | --- | --- | --- |
|  |  | Helpline | Urgent Health professional support (urgent online counselling) | Contact information for incident reporting (domestic abuse/violence ) |
| **1** | **Estenarh** |  |  |  |
| **2** | **Shezlong** | **YES** |  |  |
| **3** | **O7 Therapy** |  |  |  |
| **4** | **Labayh** |  | **YES** | **YES** |
| **5** | **Ayadi** | **YES** |  |  |
| **6** | **Tetaman** |  |  | **YES** |
| **7** | **AlMorshed** |  |  |  |
| **8** | **Mind** |  | **YES** |  |
| **9** | **Faserly** |  |  |  |
| **10** | **Daeim** |  |  |  |
| **11** | **Cura** |  |  |  |
| **12** | **Sanar** |  |  |  |
| **13** | **Akhtibar Alshakhsia Alnarjisia** |  |  |  |
| **14** | **Alruqayat Alshareia** |  |  |  |
| **15** | **Alsiha Alnafsia** |  |  |  |
| **16** | **Alsiha Alnafsia w aleaqlia** |  |  |  |
| **17** | **Daliluk Alnafsi** |  |  | **YES** |
| **18** | **Tawkidat** |  |  |  |
| **19** | **Nafas** |  |  |  |
| **20** | **Tuhoon** |  |  |  |
| **21** | **Kun Being** |  |  |  |
| **22** | **Tawazon** |  |  |  |
| **-** | **Smiling Mind** | **YES** |  |  |
| **-** | **ReachOut WorryTime** | **YES** |  |  |
| **-** | **HeadGear** | **YES** |  |  |
| **-** | **MoodMission** | **YES** |  |  |
